# Infrared‐Microwave Compatible Stealth Transparent Metasurface With Digital Camouflage and Hybrid Absorption‐Scattering

**DOI:** 10.1002/nap2.70156

**Published:** 2026-06-15

**Authors:** Dan Liu, Yanzhao Wang, Kefeng Ji, Huanhuan Gao, Sihong Zhou, Tong Liu, He‐Xiu Xu

**Affiliations:** ^1^ Air and Missile Defense College Air Force Engineering University Xi'an China; ^2^ Information and Navigation College Air Force Engineering University Xi'an China; ^3^ School of Energy Science and Engineering Harbin Institute of Technology Harbin China

**Keywords:** digital IR camouflage, hybrid absorption‐scattering, multispectral compatible stealth, RCS reduction, transparent metasurface

## Abstract

With the rapid advancement of detection technologies, single‐band stealth strategies have become ineffective, rendering research into multispectral compatible stealth imperative. Thus, there is a pressing need for stealth solutions enabling effective simultaneous stealth performance across visible, infrared (IR) and microwave bands. Here, we propose a visible‐light transparent metasurface (MS) architecture with simultaneous digital IR camouflage and microwave radar cross section (RCS) reduction. Specifically, the composite MS consists of an IR camouflage layer (IRCL) using digital camouflage strategy with varied emissivity and an RCS reduction layer (RRL) based on hybrid absorption‐scattering mechanism. Unlike traditional IR‐radar compatible stealth MS relying on IR low‐emissivity and microwave absorption, this design adopts digital IR camouflage mechanism for non‐uniform emissivity backgrounds. Moreover, the hybrid absorption‐scattering overcomes intrinsic drawback (enhanced IR radiation by heat accumulation) of pure absorption. For feasibility verification, a sample was fabricated and experimentally characterised. Experimental results indicate that the metadevice achieves an IR camouflage in 3–14 μm, a 10 dB RCS reduction within 7.5–16.5 GHz and a high visible‐light transmittance up to 55.3% and thus offers a lightweight and integrated solution for multispectral stealth applications.

## Graphical abstract

Traditional metasurfaces suffer from the drawbacks of pure microwave absorption and low IR emissivity stealth, namely failing to match complex ambient backgrounds with varying IR emissivity and generating excess heat accumulation (enhanced IR thermal radiation). To address these issues, we propose a multi‐spectrum compatible camouflage metasurface architecture via digital IR emissivity and hybrid microwave absorption‐scattering, realizing IR camouflage against diverse backgrounds, RCS reduction within wide incident angles, and effective suppression of heat accumulation.

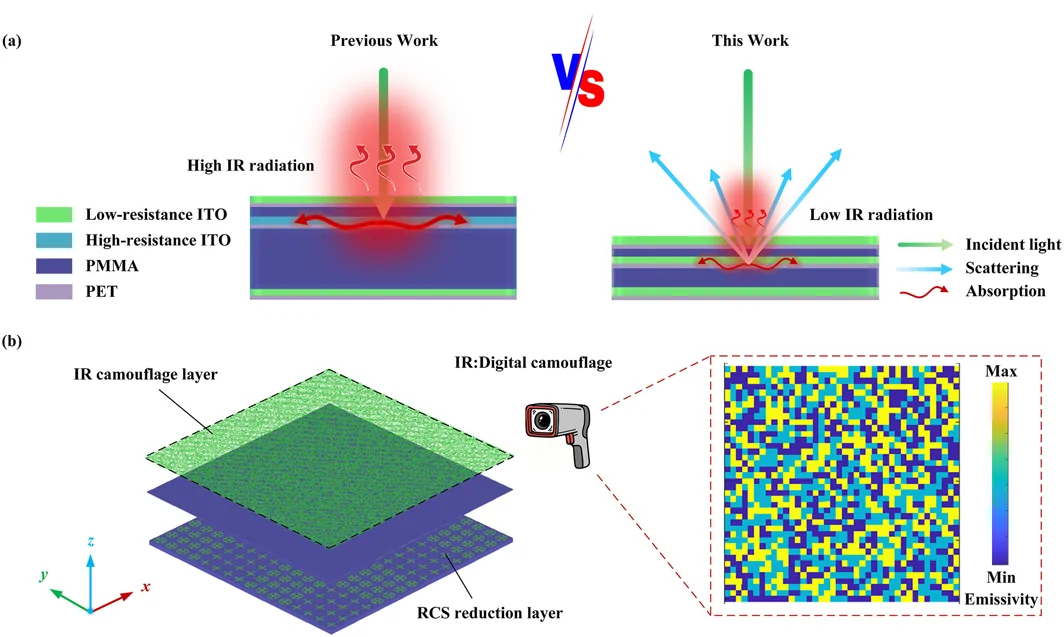

## 1 Introduction

With the rapid evolution of detection technologies in modern battlefields, single‐band stealth is inadequate in countering multi‐dimensional reconnaissance threats [1, 2, 3, 4, 5, 6]. Consequently, multispectral compatible stealth has become a fundamental requirement for survivability of military communication equipment, with infrared (IR)‐radar dual‐band compatible stealth emerging as a key challenge [7, 8, 9, 10]. From the perspective of detection principles, IR detection systems identify targets by capturing thermal radiation differences between targets and backgrounds [11, 12, 13]. For stealth targets such as aircraft, the IR thermal radiation from components such as engines and nozzles is higher than environmental background due to elevated temperatures. Therefore, low‐emissivity materials are generally adopted for IR stealth to suppress detectable thermal signals [14, 15, 16]. By contrast, radar detection relies on electromagnetic (EM) wave scattering signals to locate targets [17, 18, 19]. Thus, radar stealth technology aims to reduce the radar cross section (RCS) of targets to effectively suppress microwave signals and avoid detection [20, 21, 22]. The technical requirements of IR‐radar dual‐stealth are both independent and mutually coupled. Consequently, how to realise collaborative optimisation of IR and radar dual‐band performance on the same carrier has become a major technical challenge in stealth technology [23, 24].

To date, extensive efforts have been devoted to IR‐radar stealth compatibility in materials science, with relevant studies developing hetero‐dimensional structural materials [25], multi‐layer films [26], phase‐change material‐based adaptive coatings [27] and composite ceramic absorbers [28] to achieve multi‐band stealth performance via different material combinations. Nevertheless, these schemes still suffer from opaqueness in visible spectrum for employed materials, inability to precisely tailor EM parameters and complicated fabrication processes, which hinder their large‐scale practical applications [29]. Recently, metamaterials, leveraging their unique electromagnetic regulation capabilities, have been widely applied in the field of multispectral compatible stealth as a promising solution for these issues [30, 31, 32, 33, 34, 35, 36, 37, 38, 39, 40]. Relevant studies have developed diverse metasurface designs combining patterned films and frequency selective surfaces (FSS) [41], bioinspired bilevel structures [42], asymmetric composite structures [43] and hierarchical architectures [44] to achieve multispectral stealth via functional layer integration and structural design. However, most existing studies adopt the combination IR low‐emissivity and pure microwave absorption shown in Figure 1a. This pure microwave absorption mechanism is prone to heat accumulation [46], which causes an increase in target temperature. Based on the Stefan–Boltzmann law *E* = σ*εT* ^4^, twhere *E* is the radiant exitance (W/m^2^), *σ* = 5.68 × 10^−8^W·m^−2^ K^−4^ is the Stefan–Boltzmann constant, *ε* and *T* are the IR emissivity and temperature of the target, respectively, the increase in *T* has a dramatic effect on IR thermal radiation. Meanwhile, such low‐emissivity IR stealth designs also fail to match complex ambient backgrounds with varying IR emissivity.

**FIGURE 1:**
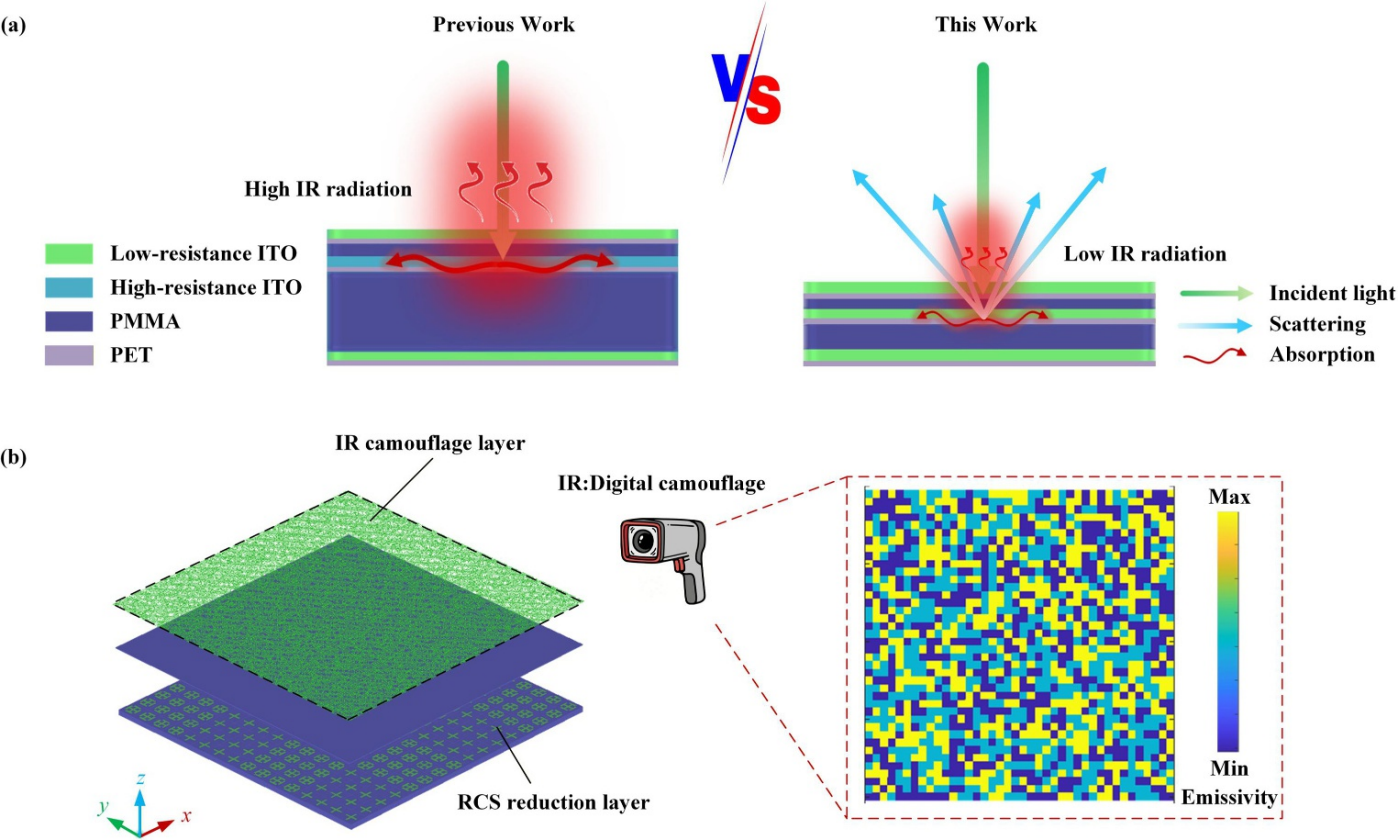
Proposed stealth architectures and MS structure. (a) Comparison of multispectral compatible stealth MS architectures between this work and previous work. The conventional structure adopts a pure microwave absorption mechanism with high‐resistance (80, 100, 300 Ω/sq and even higher) [5, 43, 45] ITO, which enhances IR thermal radiation (left panel). This work employs hybrid absorption‐scattering mechanism, achieving microwave RCS reduction with only low‐resistance (6 Ω/sq) ITO and thus effectively reducing IR thermal radiation induced by heat accumulation (right panel); (b) Architectures of designed dual‐layer MS (left panel) and IR digital camouflage effect under an IR thermal imager (right panel).

To address the above challenges, we propose a dual‐layer MS architecture composed of an infrared camouflage layer (IRCL) and a radar cross‐section reduction layer (RRL). Initially, indium tin oxide (ITO) is chosen as core functional material, which endows MS with high visible‐light transmittance via its transparency characteristic and microwave absorption capability due to its inherent loss. Subsequently, the IRCL modulates target's IR emissivity via discrete “pixel” elements, which minimises IR radiation boundary difference between target and its surroundings, thus realising digital IR camouflage. Ultimately, the RRL employs hybrid absorption‐scattering mechanism by two elements with identical absorption and 180° phase difference in a checkerboard arrangement. This strategy thereby simultaneously achieves RCS reduction and reduces IR signature by mitigating heat accumulation via microwave scattering mechanism. In practice, this synergistic dual‐layer design addresses key limitations of conventional multispectral compatible stealth MSs, providing promising applications prospects for stealth facilities and surveillance systems operating in optical windows.

## 2 Element and MS Design

To achieve compatible stealth across IR, radar and visible light, the MS is designed as a dual‐layer architecture, which is composed of three ITO layers sandwiched by two polymethyl methacrylate (PMMA) dielectric spacers, with ITO patterned on polyethylene terephthalate (PET) substrates, as shown in Figure 1b. Specifically, the ITO patterns comprise a top IRCL of square patches with varying filling ratios, a middle RRL of cross‐shaped (element A) and Jerusalem‐shaped (element B) elements in a checkerboard arrangement and a bottom ground plane. In the following, we will conduct detailed numerical design and analyse EM characteristics of IRCL, RRL and resulting MS to systematically unveil and approve the operation principle of proposed stealth architecture.

### 2.1 Numerical Design of IRCL Element

To reduce IR emissivity for better stealth performance, IRCL employs 6 Ω/sq ITO films with low emissivity [43]. Effective relative permittivity of ITO films in IR band can be accurately characterised via Drude mode [45, 47].

(1) $$\varepsilon \left(\omega \right)=\varepsilon_{b}-\frac{\omega_{p}^{2}}{\omega \left(\omega +i\omega_{c}\right)}$$

Here the background permittivity is $\varepsilon_{b}$ = 3.9, the plasma frequency is $\omega_{p}$ = 461 THz and the collision frequency is $\omega_{c}$ = 28.7 THz. Thus, the real part of permittivity is negative, which endows ITO with high reflectivity below $\omega_{p}$. To verify this, we simulated EM characteristics of ITO based on Equation (1) via CST Studio Suite commercial software in Figure 2b. The simulated results show an emissivity as low as 0.12 and a reflectivity of nearly 0.88 in 3–14 μm, which aligns well with theoretical analysis and demonstrates excellent IR suppression performance. In light of this, we design IR elements with square ITO patterns of different widths *a.* The target emissivity is precisely modulated by tuning filling ratio via *a* and can be approximated by following empirical formula [48].

(2) $$\varepsilon ={\;\varepsilon }_{\text{ITO}}{\times f}_{\text{ITO}}+\varepsilon_{\text{PET}}{\times f}_{\text{PET}}$$

Herein, $\varepsilon_{\text{ITO}}$ and $\varepsilon_{\text{PET}}$ denote IR emissivity of ITO and PET, respectively. $f_{\text{ITO}}$ and $f_{\text{PET}}$ represent their respective filling ratios, satisfying $f_{\text{ITO}}$+$f_{\text{PET}}$ = 1. Given that $\varepsilon_{\text{ITO}}$ < $\varepsilon_{\text{PET}}$, $\varepsilon_{\text{PET}}=0.9$, it is necessary to increase the filling ratio of a square patch to achieve low IR emissivity. Furthermore, for quantitative analysis, we performed numerical calculations of emissivity of IR elements with different *a* in Figure 2c. As can be seen, the average emissivity decreases with increasing *a* and falls below 0.3 when *a* > 0.85. Thus, a larger value of *a* is required to realise effective IR stealth performance.

**FIGURE 2:**
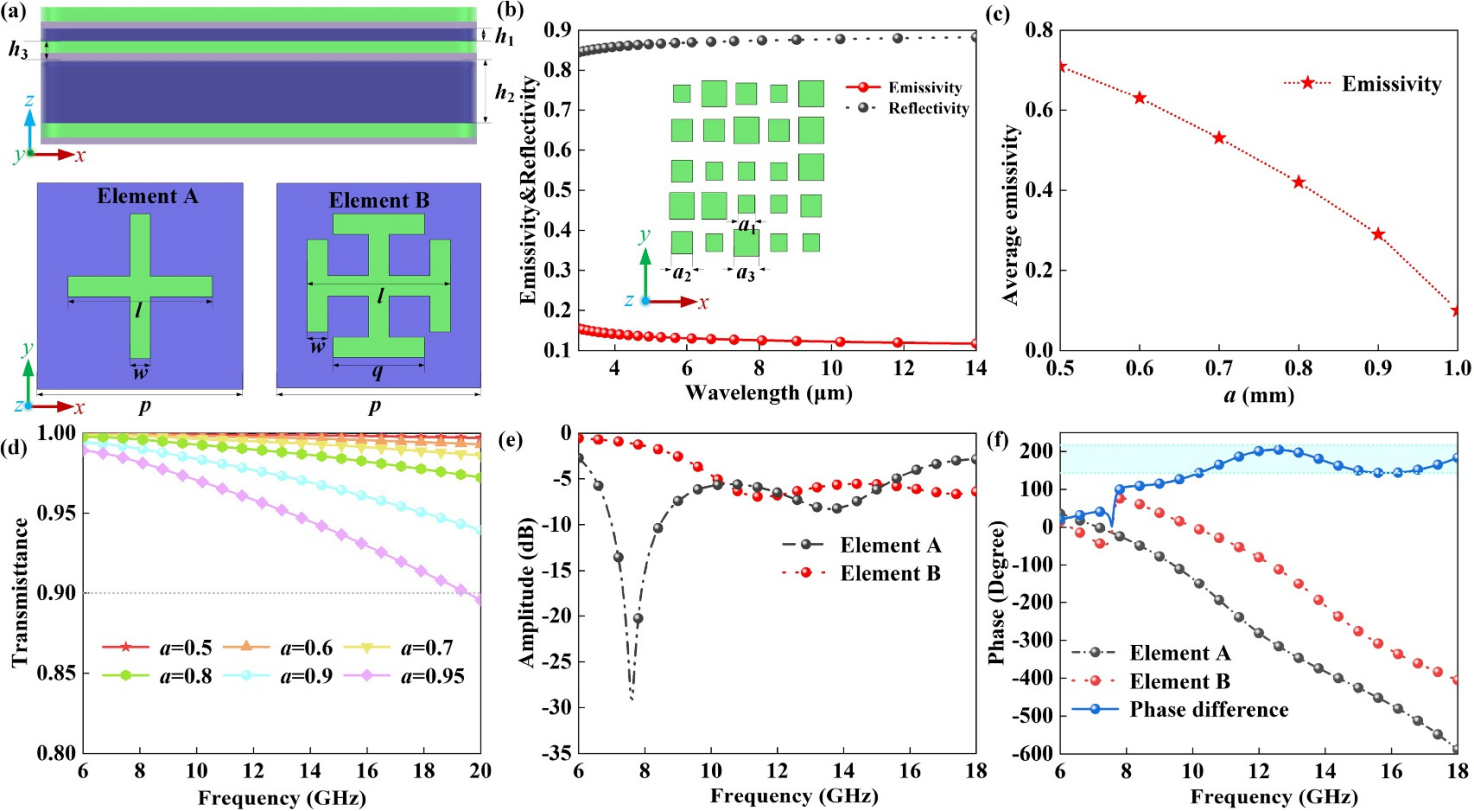
Layout and EM characterisation of elements in IR and microwave bands. (a) Side view of the whole structure (top panel) and top view of microwave element A, B in the middle layer (bottom panel), where *h*_1_ = 0.5 mm, *h*_2_ = 3 mm, *h*_3_ = 0.05 mm, *l* = 7.5 mm, *w* = 1 mm, *q* = 4.5 mm and *p* = 10 mm; (b) Simulated emissivity and reflectivity of ITO in 3–14 μm, with inset figure showing the layout of IR elements; (c) Numerically calculated average emissivity in 3–14 μm and (d) simulated microwave transmissivity within 6–20 GHz of IR elements with different *a*; (e) Simulated reflection amplitudes and (f) phase response of bare microwave elements within 6–18 GHz.

To achieve synergistic IR stealth and microwave transmission, IRCL employs a capacitive FSS with low‐pass filtering characteristics. The resonant frequency of FSS is determined as *f* = *c*/(2*na*) according to standing wave resonance theory [49]. Here, *n* represents the refractive index of dielectric substrate and *c* is the speed of light in vacuum. Obviously, a decrease in the geometric parameter *a* increases the resonant frequency, thereby enhancing transmittance of low‐frequency signals. To verify this, we simulated microwave transmittance of IR elements with different *a* within 6–20 GHz in Figure 2d. Apparently, the microwave transmittance decreases with increasing *a* and it remains above 0.9 when *a* < 0.95, which is basically consistent with theoretical derivation. Thus, a smaller value of *a* is required to enable effective microwave transmission to RRL.

Hence, considering IR emissivity and microwave transmittance comprehensively, we selected three basic IR elements with emissivity of 0.65, 0.4 and 0.3, corresponding to patch widths of *a*_1_ = 0.56, *a*_2_ = 0.79 and *a*_3_ = 0.87 and filling ratios of 31%, 62% and 76%, respectively. These three “pixel” elements thus achieve a balanced performance between low IR emissivity and high microwave transmittance, laying a foundation for subsequent digital camouflage metadevice design.

### 2.2 Design and EM Characteristics of RRL Element

To achieve RCS reduction for microwave stealth, we designed an RRL that achieves absorption and scattering simultaneously and further implemented joint optimisation of reflection amplitude and phase of elements. Specifically, based on the principle of intensity cancellation, we designed elements A and B as depicted in Figure 2a. For theoretical derivation, the total electric field intensity (*E*_total_) can be expressed as $E_{\text{Total}}$ = $E_{A}$ + $E_{B}$ = ${\;A}_{1}e^{j\varphi_{1}}$ + $A_{2}e^{j\varphi_{2}}$. Here, *A*_1_, *A*_2_ and *φ*_1_, *φ*_2_ are magnitudes and phases of reflected electric field vectors of *E*_A_, *E*_B_ from the two elements, respectively. The intensity cancellation condition is satisfied with *E*_total_ ≈ 0 when *A*_1_ = *A*_2_ and their phase difference is within 180° ± 37°. This can be achieved with a checkerboard arrangement of the two elements, thereby enabling microwave stealth performance. Accordingly, Figure 2e,f presents simulated EM characteristics of the two bare microwave elements after parameter optimisation in CST Studio Suite. Evidently, simulated results exhibit a phase difference of 180° ± 37° within 10–18 GHz. Meanwhile, a certain degree of absorption is induced by inherent ohmic loss of ITO in the microwave band, keeping the identical reflection amplitude of the two elements at approximately −7 dB. Thus, the two bare microwave elements fulfill the intensity cancellation condition, achieving both absorption and diffusive scattering.

To investigate the absorption performance of the two proposed microwave elements, numerical simulations were conducted to evaluate the absorptivity of elements A and B at various incident angles. As shown in Figure 3a and 3b which depicts absorptivity spectra from 8 to 20 GHz for incident angles between 0° and 60°, each of the two elements maintain almost consistent absorptivity across all incident angles, indicating good wide‐angle stability. Minor variations in absorptivity are only observed in the 14–18 GHz frequency band when the incident angle exceeds 30° and each element presents a well‐defined absorption peak at 13.5 and 11.5 GHz, respectively. To further reveal the underlying absorption mechanisms, the electric field distributions and surface current distributions of the two elements at their respective resonant frequencies are presented in Figure 3c–f. For element A, electric fields concentrate at the vertical arm ends and surface currents on the horizontal arms at 11.5 GHz, forming a fundamental dipole resonance. At 13.5 GHz, fields shift to the central vertical arm and horizontal arm inner edges, with currents enhanced at the cross intersection, corresponding to a higher‐order dipole resonance. Both modes trap electromagnetic energy in resistive ITO film, dissipating it via ohmic losses. For element B, electric fields gather at the inner corners of T‐shaped arms and surface currents form four symmetric closed loops at 11.5 GHz, exciting a quadrupole resonance. At 13.5 GHz, fields extend to the outer edges of the T‐arms and currents become more dispersed, corresponding to a dipole‐quadrupole hybrid resonance. The symmetric design of both elements makes their resonant modes insensitive to incident angles up to 60°, accounting for their excellent wide‐angle stability.

**FIGURE 3:**
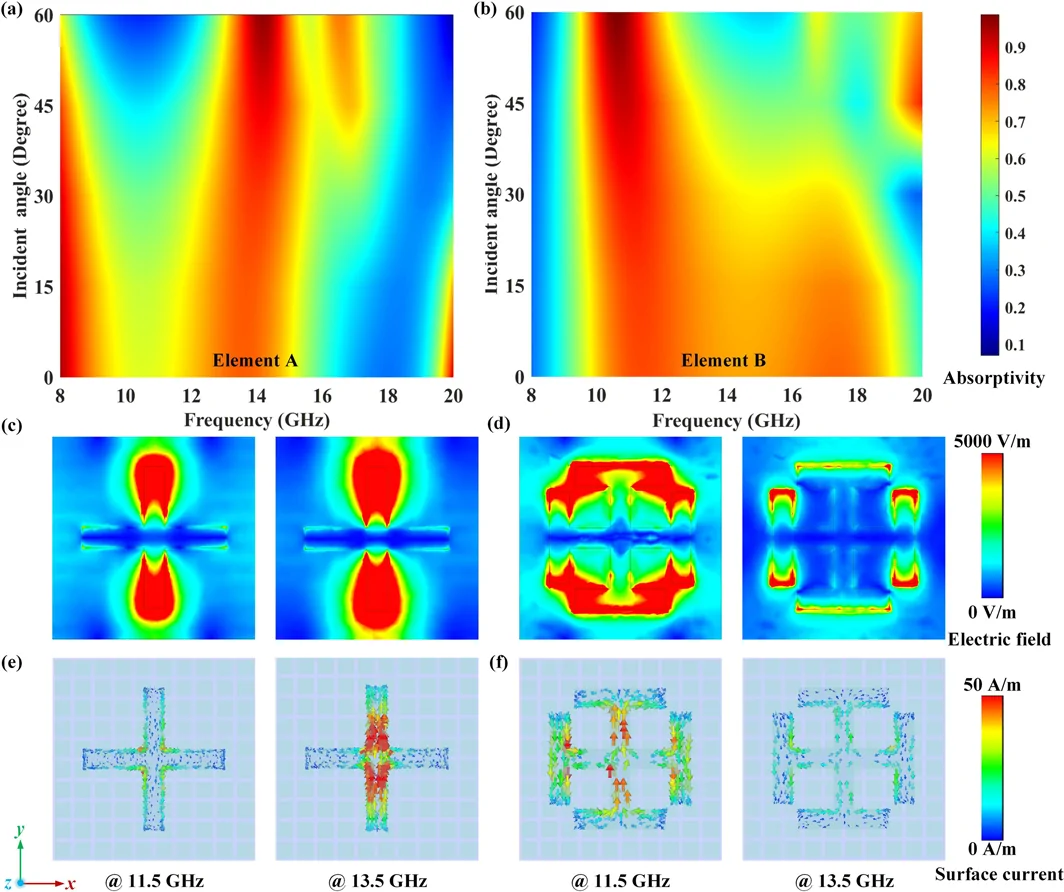
Numerical characterization of two microwave elements. (a, b) Absorption at different incident angles, (c, d) electric field and (e, f) surface current distributions at 11.5 and 13.5 GHz of elements A and B, respectively.

To investigate the effect of IRCL on RRL at microwave band, we performed numerical simulations on EM characteristics of element A and B integrated with different top patch patterns. Specifically, four cases of different patch patterns are considered with uniform (IRCL‐I) and random (IRCL‐II to IV) arrangements to individually illustrate the effects of patch size and arrangement. For one thing, we characterised the performance of conventional IR stealth architecture IRCL‐I. It features uniformly low emissivity and the corresponding patch pattern is depicted in Figure 4a. Evidently, the amplitude and phase responses hold nearly consistent trends as a change of different *a* in Figure 4b–e. For another, we design top ITO patches in different random arrangements, corresponding to digital camouflage of IRCL with distinct patterns to verify EM characteristics of the two microwave elements. Specifically, Figure 5a–c shows the layouts of three distinct random patch patterns IRCL‐II to IV and Figure 5d–i plots their corresponding reflection amplitudes and phases. Obviously, they remain nearly constant trends across 6–18 GHz and the phase differences are all within 180° ± 37° over 10–20 GHz. Hence, the EM characteristics of elements in RRL are barely affected by size and arrangement of top‐layer patches, maintaining nearly identical absorption and satisfying intensity cancellation conditions. It should be noted that the EM response of integrated IRCL‐I to IV shifts towards lower frequencies due to increased overall thickness compared with that of bare microwave elements, which is beneficial to stealth operation bandwidth.

**FIGURE 4:**
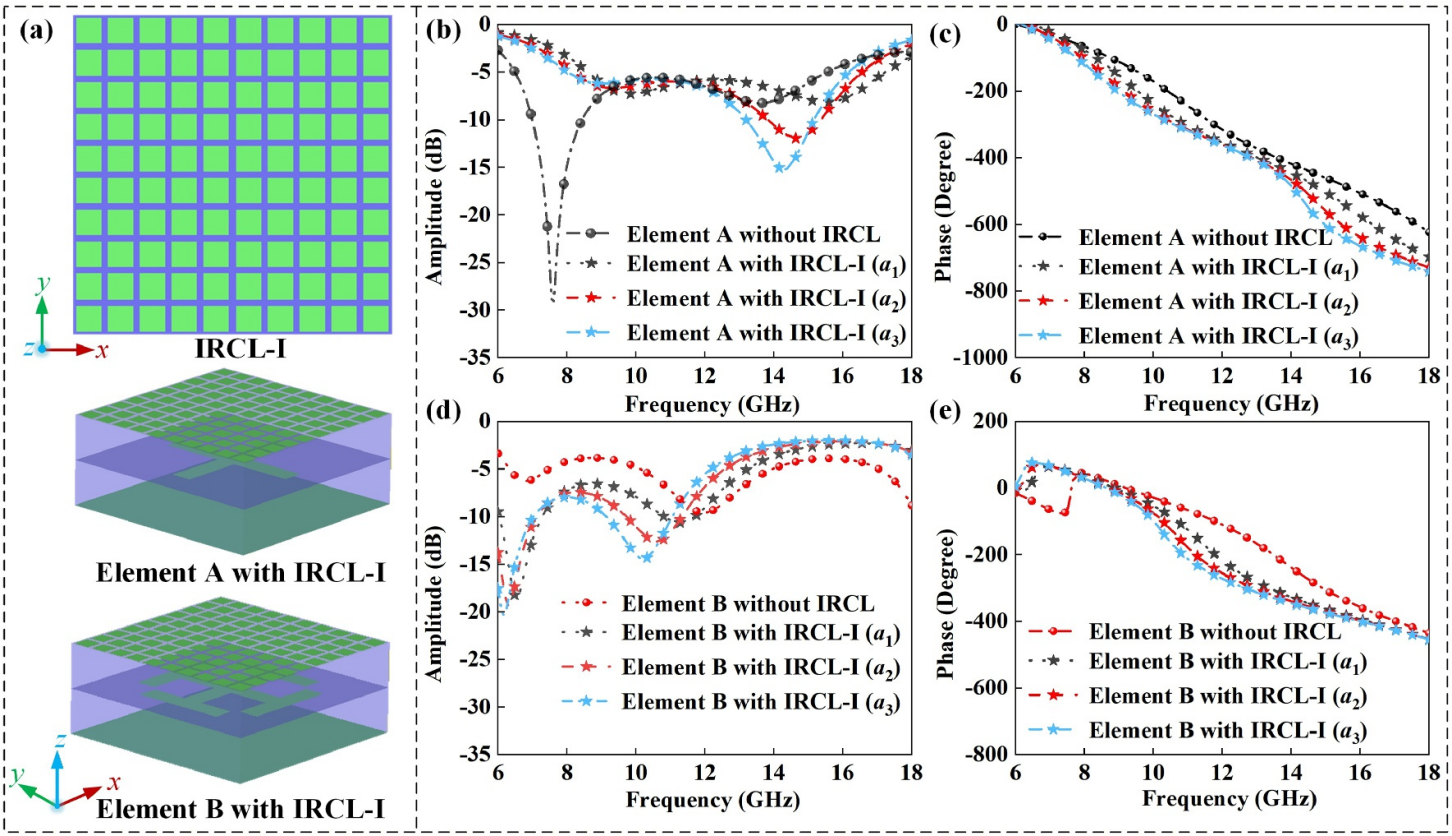
Structure and simulated EM response of MS elements in microwave band. (a) Top views of conventional IR stealth arrangement IRCL‐I with uniform patch patterns and structures of element A and B integrated IRCL‐I; Simulated (b, d) reflection amplitudes and (c, e) phases of element A and B integrated IRCL‐I with different *a*, respectively.

**FIGURE 5:**
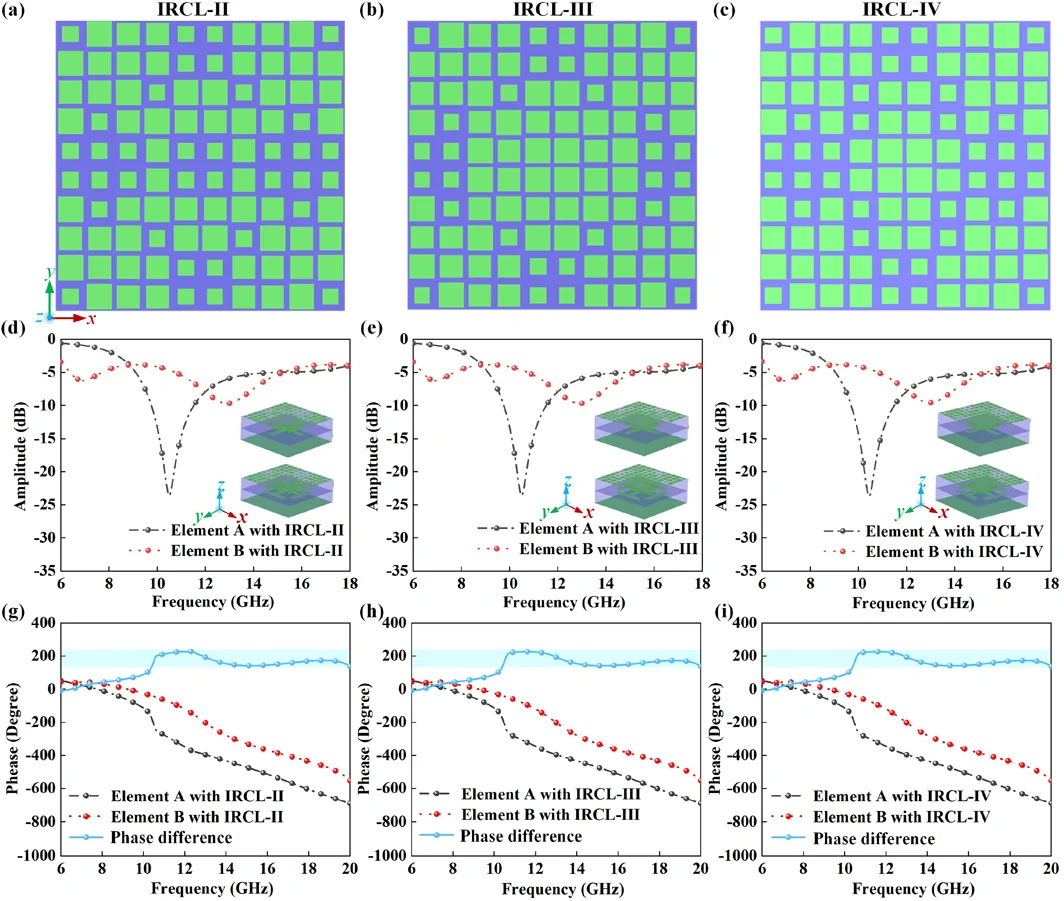
Layout and simulated microwave characteristic under diverse IRCL random patch patterns. Top views of three cases with different random patch patterns: (a) IRCL‐II, (b) IRCL‐III and (c) IRCL‐IV; (d–f) Reflection amplitudes and (g–i) phase response of the two microwave elements with IRCL‐II, IRCL‐III and IRCL‐IV, respectively.

### 2.3 Design and EM Characteristics of MS

To achieve digital IR camouflage, the selected three IR “pixel” elements of IRCL are arrayed randomly, which is based on visual psychological characteristics of human graphic, digital imaging dot matric and human visual target recognition. It can mimic the background's color, texture and fine details in the form of a digital “dot matric” on the target surface. Meanwhile, the IRCL array is randomly arranged with central symmetry to minimise the impact on functionality of RRL. Thus, Figure 1b only shows a schematic of a quarter of the entire array, with the remaining three‐quarters arranged symmetrically in accordance with this layout.

To design a microwave array with optimal RCS reduction performance, simulated 3‐D far‐field scattering patterns based on CST time‐domain solver were performed on three types of arrays shown in Figure 6 at 10, 12 and 14 GHz. Specifically, Figure 6a shows full‐array 1 composed of element A only. Figure 6b shows full‐array 2 composed of element B only and Figure 6c shows full‐array 3 composed of subarray A (4 × 4 element A) and subarray B (4 × 4 element B) in a checkerboard arrangement. Obviously, the energy of full‐array 1 and 2 is mainly concentrated along normal direction, generating a single beam. In contrast, full‐array 3 generates four symmetrical beams under effect of intensity cancellation and its energy in normal direction is significantly lower than that of full‐array 1 and 2. Meanwhile, Figure 7a plots the RCS reduction curves of the three arrays in 6–20 GHz to further investigate their stealth performance. Apparently, under the effect of pure absorption, full‐array 1 achieves a 10 dB RCS reduction only within a narrow band of 12.5–14.5 GHz compared with a same‐size metal plate, whereas full‐array 2 fails to realise a 10 dB RCS reduction across the entire 6–20 GHz. In contrast, full‐array 3 achieves a 10 dB RCS reduction over a broad band of 9.5–20 GHz and attains a peak value of 33 dB at 11.5 GHz, thus it was selected as the final RRL array.

**FIGURE 6:**
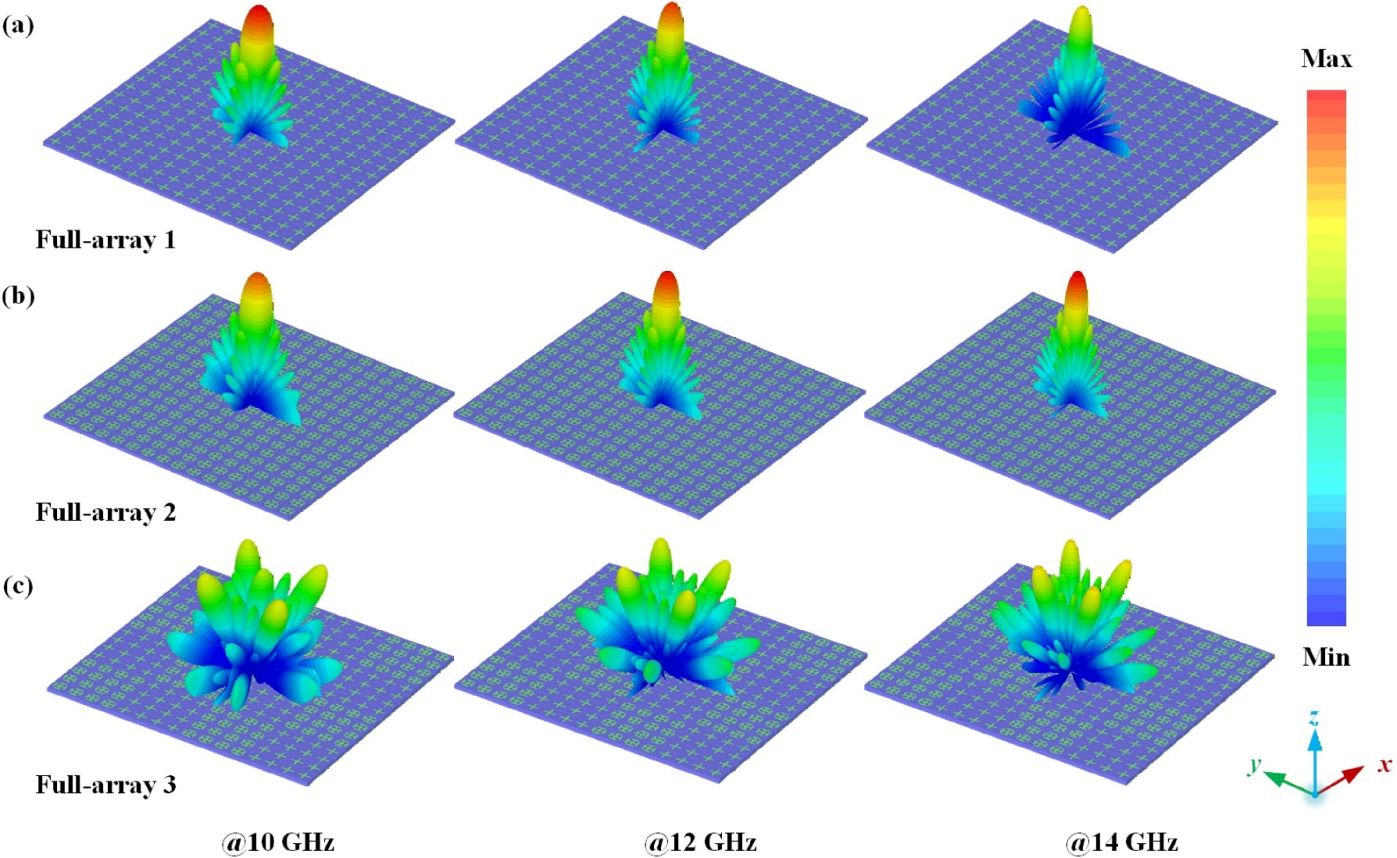
Comparison of simulated 3‐D far‐field scattering patterns of microwave layer with identical size and different arrangements at 10, 12 and 14 GHz, respectively: (a) full‐array 1 only composed of element A, (b) full‐array 2 only composed of element B and (c) full‐array 3 composed by subarray A (4 × 4 element A) and subarray B (4 × 4 element B) in a checkerboard arrangement.

**FIGURE 7:**
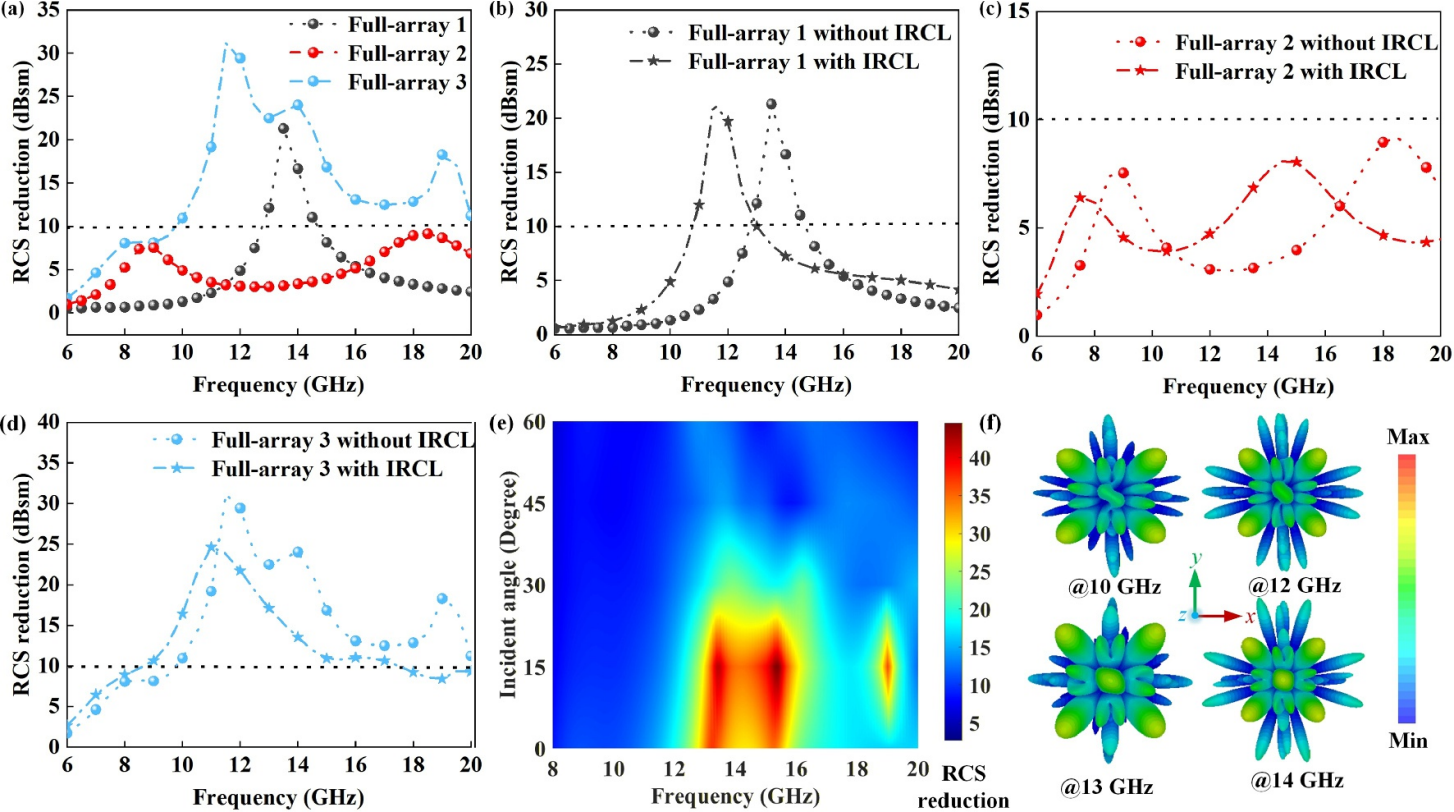
Simulated RCS spectra and 3‐D far‐field scattering patterns of metadevice. (a–d) RCS reduction comparison of (b) full‐array 1, (c) full‐array 2 and (d) full‐array 3 with and without IRCL within 6–20 GHz; (e) RCS reduction of full‐array 3 with IRCL at incident angles of 0°–60°; (f) 3‐D far‐field scattering patterns of full‐array 3 with IRCL at 10, 12, 13 and 14 GHz, respectively.

From Equation (2), IR emissivity depends only on the top‐layer material and its filling ratio, independent of design of underlying structure. Thus, only the effect of IRCL on microwave stealth performance of RRL is discussed in the following. For such a purpose, Figure 7b–d shows RCS reduction comparison curves of full‐array 1 to 3 with and without IRCL, respectively. It is evident that the RCS spectra all shift towards lower frequencies after integrating IRCL, whereas the overall trend remains consistent. It should be noted the frequency shift at higher frequencies is slightly larger than lower frequencies, as higher frequencies correspond to shorter wavelengths and thus are more sensitive to thickness fluctuations. In addition, the operation bandwidth changes from 9.5–20 GHz to 8.5–18 GHz and corresponding relative bandwidth increases slightly from 71.43% to 71.7%, indicating that the incorporation of IRCL is beneficial to microwave stealth bandwidth. Furthermore, as shown in Figure 7e, the proposed structure maintains significant RCS reduction across the entire 0°–60° incident angle range, with a maximum reduction exceeding 35 dB in the 12–18 GHz band. The RCS reduction performance exhibits excellent wide‐angle stability, which is consistent with the absorptivity characteristics of elements analyssed earlier. In addition, 3‐D far‐field scattering patterns were performed on the resulting MS based on CST time‐domain solver, with the results presented in Figure 7f. As can be seen, the metadevice still retains distinct symmetrical four‐beam patterns at 10, 12, 13 and 14 GHz, which still maintains effective performance of intensity cancellation after IRCL integration.

## 3 Experiment Validation

To verify our multispectral compatible stealth mechanism, we conducted experiments on the fabricated metadevice. The patterns were fabricated by a standard industrial nanosecond ultraviolet (UV) laser etching process on commercially sourced conductive ITO films deposited on visible‐light‐transparent PET substrates. This process enables selective removal of the ITO conductive layer without damaging the underlying flexible PET substrate, achieving a minimum pattern linewidth of ∼50 μm and an alignment accuracy better than ± 10 μm. Then, the metadevice was constructed by attaching these patterned ITO films to both sides of a commercial‐grade PMMA plate using optical‐grade pressure‐sensitive adhesive. Subsequently, the experimental characterisations were performed from three key aspects, including emissivity, IR images at different temperatures and thermal stability of the metadevice to systematically verify its IR camouflage performance. Specifically, the photograph of fabricated metadevice with a dimension of 160 × 160 × 3.65 (total thickness *h* = *h*_1_ + *h*_2_ + 3 × *h*_3_) mm^3^, Fourier transform IR spectrometer (FTIR) and corresponding experiment setup are shown in Figure 8a. Experimental results of its IR transmittance, reflectance and emissivity are presented in Figure 8b. Apparently, the emissivity is approximately 0.4 within 3–14 μm, which is consistent with average emissivity (0.3, 0.4 and 0.65) of the three selected IR elements. Furthermore, Figure 8g shows its IR images at 12°C, 35°C, 55°C and 85°C in descending order from top to bottom, all captured after the heating platform stabilised at set temperatures and reached thermal equilibrium. The topmost image corresponds to the condition of winter room temperature 12°C and was captured with a metadevice placed on an unheated metal backplane. The radiation temperature of metal background was 3.3°C slightly lower than metadevice owing to ambient environmental temperature effects. Nevertheless, it still achieves excellent background fusion in IR images captured at this condition. Moreover, the apparent radiation temperature is significantly lower than that of background heating at temperatures 35°C, 55°C and 85°C, indicating the robust stealth capability of a metadevice against backgrounds at different temperatures. In addition, Figure 8h presents IR images of metadevice placed on a heating stage for holding time of 3, 5, 15 and 30 min, respectively. As can be seen, both radiation temperatures of metadevice and background increase to some extent during continuous heating, with the metadevice showing a lower temperature in all cases and a slower rate of increase relative to the background. Thus, our designed metadevice displays effective IR camouflage performance and excellent thermal stability.

**FIGURE 8:**
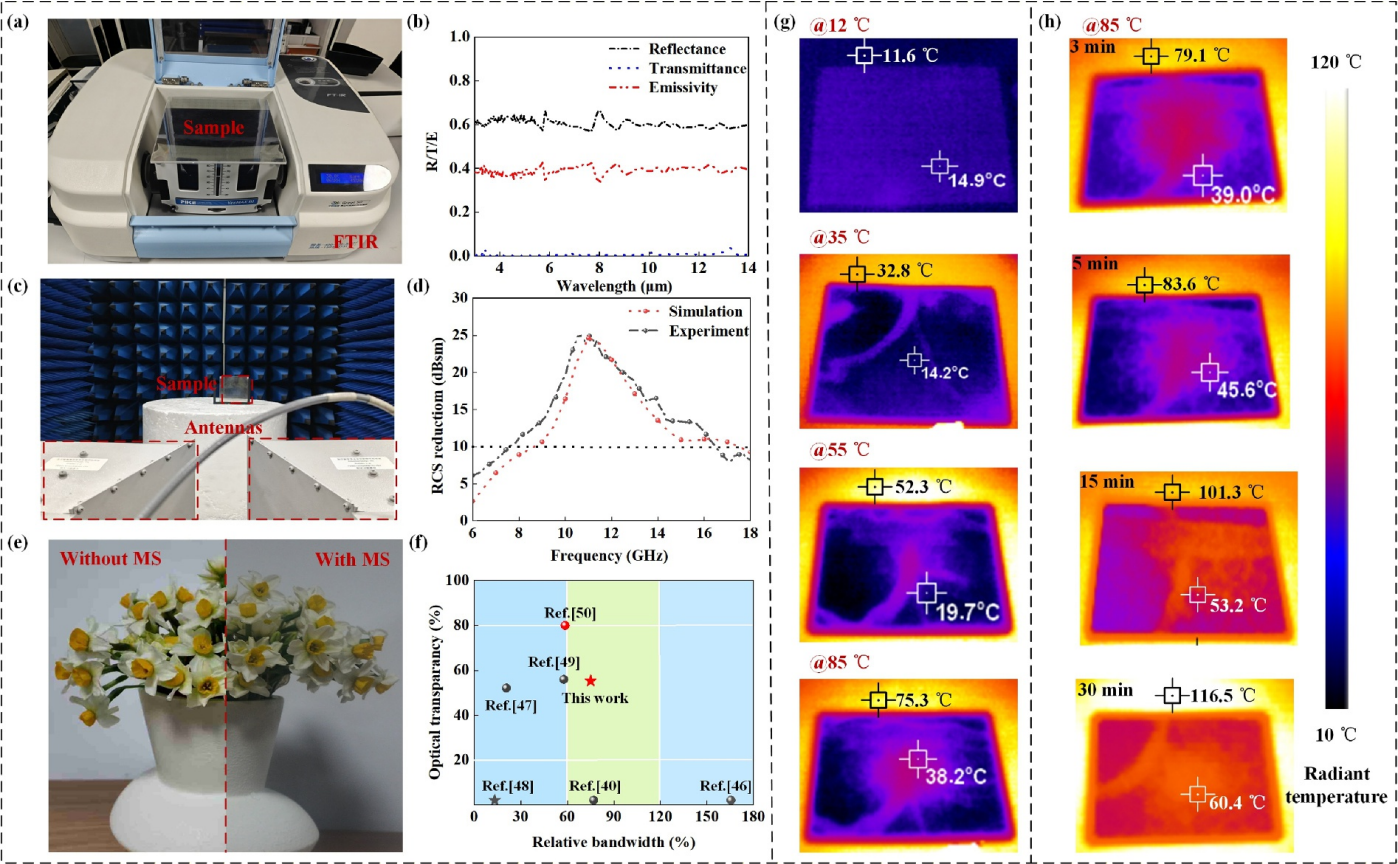
Experimental results of the fabricated metadevice. (a) FTIR setup for IR emissivity; (b) IR reflectivity, transmittance and emissivity (denoted as R, T and E, respectively) within 3–14 μm; (c) Experimental setup for RCS measurement; (d) Comparison of simulated and measured RCS reduction within 6–18 GHz; (e) Illustration of optical transmittance; (f) Stealth performance comparison between our work and other reported designs (star symbols represent IR digital camouflage schemes, circular symbols represent non‐IR digital camouflage schemes, red symbols represent hybrid absorption‐scattering mechanism and black symbols represent pure absorption mechanism); (g) Digital IR emissivity at temperatures of 12°C, 35°C, 55°C and 85°C, respectively; (h) Digital IR emissivity at 85°C with heating time of 3, 5, 15 and 30 min, respectively.

Furthermore, to verify microwave stealth performance of the metadevice, we performed far‐field RCS measurement in a microwave anechoic chamber with experimental setup shown Figure 8c. The microwave RCS spectral was recorded using a vector network analyser, with a pair of broadband horn antennas operating at 6–18 GHz employed as the transmitter and receiver. The metadevice was positioned equidistant from the pair of horn antennas. All measured electric fields scattered from metadevice are normalised to those of a metal plate with identical dimensions. Figure 8d presents measured RCS spectral of metadevice under normal incidence, which are in excellent agreement with simulated results. It should be noted that actual overall thickness of metadevice is slightly higher than designed one due to tolerances inherently introduced in manufacturing process, leading to an overall red shift of measured results towards lower frequencies. In addition, the measured 10 dB RCS reduction bandwidth spans 7.5–16.5 GHz with a relative bandwidth of 75%, indicating that fabrication errors do not compromise overall stealth performance but rather enhance microwave stealth bandwidth. To demonstrate visible‐light stealth performance, a comparison of optical images of floral bouquet was captured in Figure 8e with and without metadevice. As can be seen, there is only a slightly darker tone after placing the metadevice, whereas the floral shape, color and texture remain clearly visible. Furthermore, experimental measurements also indicate that optical transmittance of the metadevice reaches 55.3%, confirming its favorable visible‐light stealth performance.

To illustrate compatible stealth mechanisms and comprehensive performance of our architecture, Figure 8f compares key characteristics between this work and previously reported designs, with detailed parameters provided in Table 1. Evidently, our proposed architecture features a simpler structure, a broad bandwidth and favourable visible‐light transparency. In addition, results further verify that the artificially designed ITO metadevice achieves high precision in digital IR camouflage and RCS reduction and can be rapidly and accurately customised according to on‐demand requirements. Moreover, it is compatible with existing metamaterial fabrication processes and thus supports large‐scale integrated manufacturing.

**TABLE 1:** Comparison of proposed metadevice with previously reported devices.

Reference	10 dB RCS reduction band [GHz]	RCS reduction mechanism	IR digital camouflage	Visible transparency	Thickness (mm)/*λ*_L_
[24]	1.96–20.72 (165.4%)	Absorption	No	No	15.5/0.1
[43]	8–18 (76.92%)	Absorption	No	No	7.5/0.2
[50]	5.2–6.4 (20.69%)	Absorption	No	52.13%	3.3/0.06
[51]	10.5–12 (13.21%)	Absorption	Yes	No	/
[52]	7.4–13.4 (57.7%)	Scattering	No	56%	3.35/0.08
[53]	10.6–19.4 (58.6%)	Absorption + scattering	No	80%	1.6/0.06
This work	7.5–16.5 (75%)	Absorption + scattering	Yes	55.3%	3.65/0.09

## 4 Conclusion

In summary, to achieve excellent IR, radar and visible‐light compatible stealth performance, we propose a dual‐layer MS architecture composed of an IRCL and a RRL. For IR stealth, the metadevice is engineered with digital emissivity (0.3, 0.4 and 0.65) camouflage mechanism to conceal targets in cluttered backgrounds, obscure IR signal features and impede detection. Its low average emissivity (0.4) in 3–14 μm also fully satisfies IR stealth performance requirements. For microwave stealth, the metadevice employs absorption‐scattering hybrid mechanism and experimental validation confirms a 10 dB RCS reduction across 7.5–16.5 GHz with a relative bandwidth of 75%. Moreover, it adopts low‐resistance, visible‐transparent ITO‐coated PET films and PMMA dielectric, yielding a measured visible transmittance of 55.3%. Overall, we offer an effective metadevice architecture for multispectral compatible stealth, showing significant potential for future integration into lightweight, optical window‐equipped stealth platforms in military and aerospace engineering.

## Author Contributions

**Dan Liu:** investigation, conceptualization, writing – original draft, methodology, software, data curation, formal analysis, writing – review and editing. **Yanzhao Wang:** investigation, software, data curation. **Kefeng Ji:** formal analysis, visualization, validation. **Huanhuan Gao:** investigation, writing – review and editing. **Sihong Zhou:** writing – review and editing. **Tong Liu:** writing – review and editing. **He‐Xiu Xu:** funding acquisition, conceptualization, supervision, project administration, resources, writing – review and editing, validation.

## Conflicts of Interest

The authors declare no conflicts of interest.

## Data Availability

Data sharing is not applicable to this article as no datasets were generated or analyssed during the current study.
